# Effects of Alzheimer-Like Pathology on Homocysteine and Homocysteic Acid Levels—An Exploratory In Vivo Kinetic Study

**DOI:** 10.3390/ijms22020927

**Published:** 2021-01-18

**Authors:** Hendrik Nieraad, Natasja de Bruin, Olga Arne, Martine C. J. Hofmann, Robert Gurke, Dominik Schmidt, Marcel Ritter, Michael J. Parnham, Gerd Geisslinger

**Affiliations:** 1 Fraunhofer Institute for Translational Medicine and Pharmacology ITMP, Theodor-Stern-Kai 7, 60596 Frankfurt am Main, Germany; Natasja.Debruin@itmp.fraunhofer.de (N.d.B.); olga.arne@googlemail.com (O.A.); Martine.Hofmann@itmp.fraunhofer.de (M.C.J.H.); robert.gurke@itmp.fraunhofer.de (R.G.); Dominik.Schmidt@ime.fraunhofer.de (D.S.); Marcel.Ritter@itmp.fraunhofer.de (M.R.); mike.j.parnham@gmail.com (M.J.P.); geisslinger@em.uni-frankfurt.de (G.G.); 2 Pharmazentrum Frankfurt/ZAFES, Institute of Clinical Pharmacology, Goethe University, Theodor-Stern-Kai 7, 60590 Frankfurt am Main, Germany

**Keywords:** hyperhomocysteinemia, vitamin B deficiency, alzheimer disease, disease models, animal

## Abstract

Hyperhomocysteinemia has been suggested potentially to contribute to a variety of pathologies, such as Alzheimer’s disease (AD). While the impact of hyperhomocysteinemia on AD has been investigated extensively, there are scarce data on the effect of AD on hyperhomocysteinemia. The aim of this in vivo study was to investigate the kinetics of homocysteine (HCys) and homocysteic acid (HCA) and effects of AD-like pathology on the endogenous levels. The mice received a B-vitamin deficient diet for eight weeks, followed by the return to a balanced control diet for another eight weeks. Serum, urine, and brain tissues of *App^NL-G-F^* knock-in and C57BL/6J wild type mice were analyzed for HCys and HCA using LC-MS/MS methods. Hyperhomocysteinemic levels were found in wild type and knock-in mice due to the consumption of the deficient diet for eight weeks, followed by a rapid normalization of the levels after the return to control chow. Hyperhomocysteinemic *App^NL-G-F^* mice had significantly higher HCys in all matrices, but not HCA, compared to wild type control. Higher serum concentrations were associated with elevated levels in both the brain and in urine. Our findings confirm a significant impact of AD-like pathology on hyperhomocysteinemia in the *App^NL-G-F^* mouse model. The immediate normalization of HCys and HCA after the supply of B-vitamins strengthens the idea of a B-vitamin intervention as a potentially preventive treatment option for HCys-related disorders such as AD.

## 1. Introduction

The endogenous and non-proteinogenic amino acid homocysteine (HCys) has obtained increasing attention during the last decades due to the potential contribution to versatile pathologies. It is part of the one-carbon metabolism, which physiologically enables diverse methylation reactions such as during the synthesis of nucleic acids, proteins, neurotransmitters [1]. Besides its remethylation to methionine, that is dependent on the adequate supply of vitamin B12 and folate, HCys is metabolized to cysteine in a vitamin B6-dependent manner in the transsulfuration pathway [1,2]. In contrast to its metabolization, the urinary excretion of unchanged HCys is minimal [3]. Lack of the aforementioned B-vitamins as important enzymatic cofactors causes tissues to export HCys into plasma [3]. Elevated levels of HCys in the blood are referred to as hyperhomocysteinemia. As summarized by Cohen and colleagues, hyperhomocysteinemia can arise for different reasons, including a lack of relevant B-vitamins [4]. At present, a hyperhomocysteinemic state is suggested to contribute to and exacerbate different human disorders, covering the whole range of cardiovascular diseases, cancer and neurologic disorders such as Alzheimer’s disease (AD) [1,5]; the latter is also the focus of the current study. Dementia affects more than 50 million people today [6]. Especially AD, which accounts for most dementia cases [7], still lacks appropriate medical interventions to treat or prevent the ongoing neurodegenerative processes. In the current study, a knock-in mouse model for AD was used, which simulates amyloid-β (Aβ) pathology that is one of the most prominent hallmarks of the complex disease [8,9]. Saido and colleagues generated the *App^NL-G-F^* knock-in (KI) model and overcame problems of transgenic AD models based on massive amyloid precursor protein (APP) overexpression [10]. The disturbed Aβ metabolism in these mice results in aggressive Aβ amyloidosis and deposition in the brain from 2 months on. The early-stage plaque deposition has also been confirmed recently by others [11].

HCys is thought to be linked to the pathology of AD through direct and indirect neurotoxic mechanisms, as reviewed recently [12]. Hyperhomocysteinemia might not only be associated with AD [13,14], but also causally linked to AD pathology [15,16,17,18,19,20] and vascular contributions to dementia [20]. B-vitamin treatment has been shown to slow cognitive decline in subjects with elevated HCys levels [21] and therefore, may provide a preventative approach [22,23]. However, because of ambiguous findings in different studies [24,25], especially the role of B-vitamins as a potential preventative approach in cognitive disorders, there is still a lack of agreement on this topic [26,27,28,29]. In the context of neurodegenerative investigations, it is crucial that not only blood HCys but also cerebral levels are taken into consideration. HCys reaches cerebral tissues by crossing the blood-brain barrier [30] and also disrupts it [31,32]. Detrimental effects on the blood-brain barrier are exerted, for example, by excitotoxic species [33,34] such as homocysteine and homocysteic acid (HCA). The oxidative metabolite HCA is suggested to be more neurotoxic than HCys itself [35,36,37], and therefore, we assessed HCA levels as well.

In previous work from our group, we observed normalization of HCys and HCA serum levels in *App^NL-G-F^* mice after they had received control chow for only a short period of time in the IntelliCage behavioral testing system [38]. The current exploratory study attempts to provide better comprehension of the long-term kinetics, i.e., increase in HCys and HCA in mice fed a special diet deficient in B-vitamins and the expected normalization after the animals return to a normal diet. By frequently collecting mouse samples and analyzing these via LC-MS/MS, we aimed to elucidate the following central questions:

1. Is there a genotype effect in the hypothesized elevation of HCys and HCA levels in the *App^NL-G-F^* knock-in mouse model for AD compared to C57BL/6J wild type mice?
2. Are there sex-specific differences between male and female mice?
3. What is the availability of HCys and HCA in cerebral tissues?
4. Are there correlations in HCys and HCA between the different biological matrices?

## 2. Results

Body condition scores were not affected by genotype or sex. Differences were observed in the weights: male mice weighed significantly more than female mice and KI mice weighed slightly more in comparison to WT mice.

### 2.1. Serum Analysis

**Figure 1:**
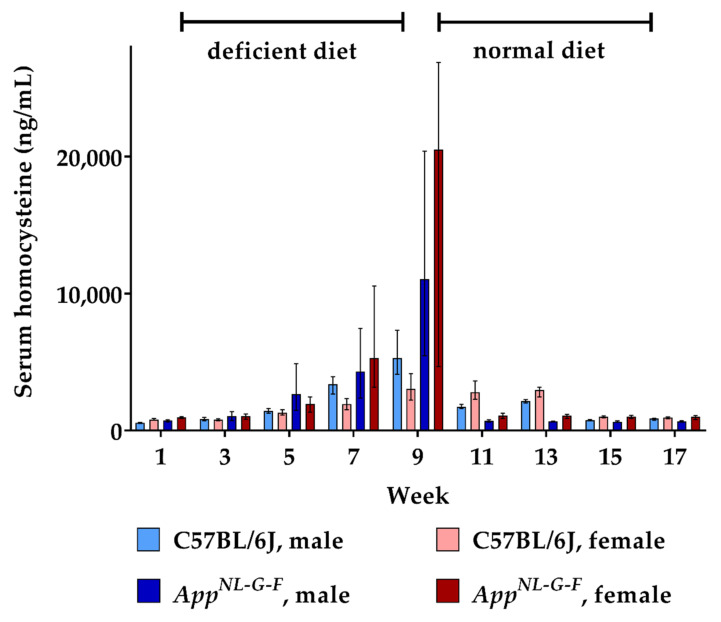
Serum homocysteine levels in C57BL/6J wild type and *App^NL-G-F^* knock-in mice, data are depicted for males and females separately for experimental week 1-17: baseline measurement (w. 1), diet deficient in vitamin B6, B12 and folate (for 8 w.), balanced normal chow (for 8 w.); all serum samples were analyzed using a combination of liquid chromatography with tandem mass spectrometry and statistically tested non-parametrically using the Kruskal-Wallis test; data are presented as median ± interquartile range (IQR); extreme outliers beyond 3xIQR have been excluded.

**Figure 2:**
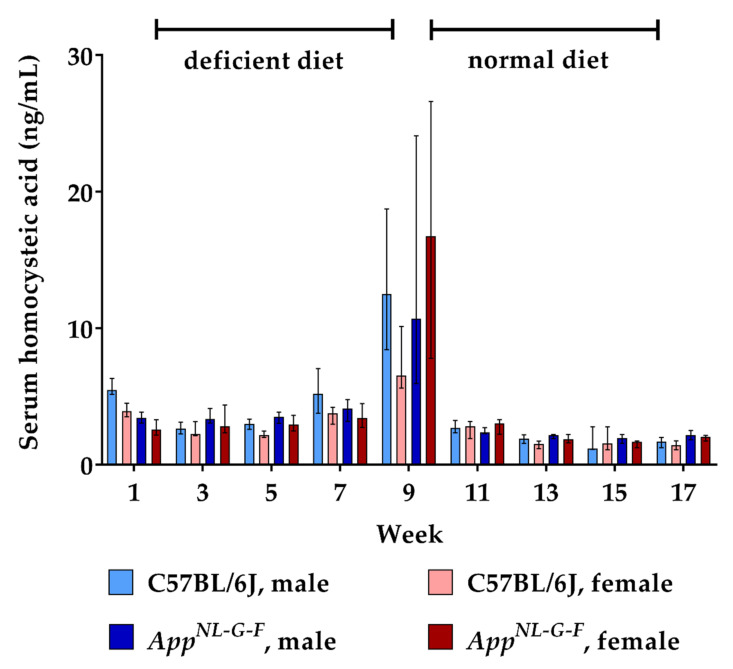
Serum homocysteic acid levels in C57BL/6J wild type and *App^NL-G-F^* knock-in mice, data are depicted for males and females separately for experimental week 1–17: baseline measurement (w. 1), diet deficient in vitamin B6, B12 and folate (for 8 w.), balanced normal chow (for 8 w.); all serum samples were analyzed using a combination of liquid chromatography with tandem mass spectrometry and statistically tested non-parametrically using the Kruskal-Wallis test; data are presented as median ± interquartile range (IQR); extreme outliers beyond 3xIQR have been excluded.

During the course of the study, serum was sampled and analyzed using a validated LC-MS/MS method. The resulting data for HCys and HCA are depicted in Figure 1 and Figure 2. Consumption of the B-vitamin deficient diet caused steadily increasing serum HCys in both males and females over eight weeks of dietary intake, resulting in about 4400 ng/mL (≈33 µmol/L) mean serum HCys (male + female) in WT and 15,400 ng/mL in KI (≈114 µmol/L) at week 9. *App^NL-G-F^* KI mice developed significantly higher HCys levels on a B-vitamin deficient diet compared to C57BL/6J WT animals (week 9: *p* < 0.001). There were no sex-related effects detected in HCys levels between males and females after the onset of hyperhomocysteinemia (week 9: *p* = 0.070). Merely at baseline levels at the start (week 1: *p* < 0.001) and at the end of the study (week 17: *p* = 0.002), HCys levels were slightly higher in females in comparison to males. The return to control chow after eight weeks on the deficient diet (week 9) resulted in a rapid normalization of serum HCys. These levels took slightly longer to decrease to baseline in females. This delayed normalization was more obvious in WT mice (week 11 and 13).

Similar to HCys, serum levels of its metabolite HCA also increased due to the lack of vitamin B6, B12 and folate over eight weeks. At week 9, approximately 0.15% of HCys molecules had undergone oxidation to HCA, reaching a mean serum level of about 15 ng/mL. There were no consistent differences between WT and KI mice, nor between males and females regarding the increase in HCA levels. Following the return to the balanced control diet, serum HCA had normalized to baseline levels already at the subsequent sampling point (week 11).

### 2.2. Urine Analysis

**Figure 3:**
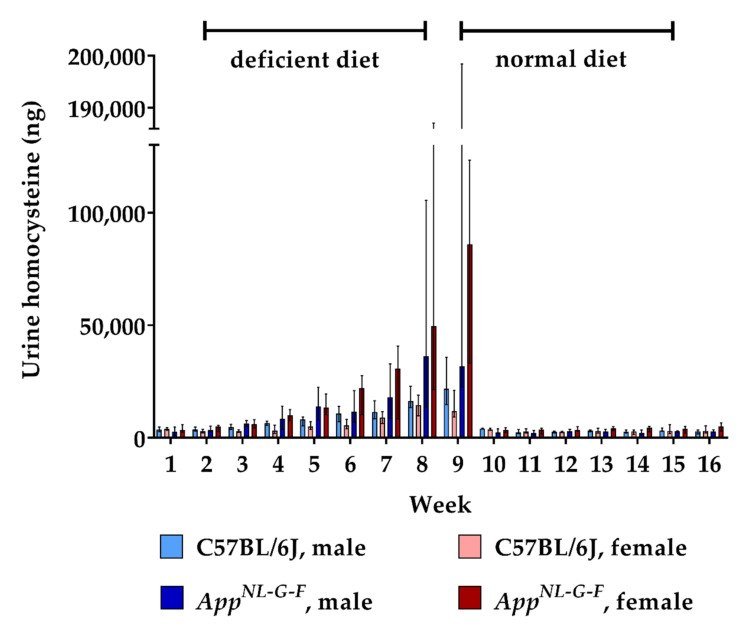
Urinary homocysteine levels in C57BL/6J wild type and *App^NL-G-F^* knock-in mice, data are depicted for males and females separately for experimental week 1-17: baseline measurement (w. 1), diet deficient in vitamin B6, B12 and folate (for 8 w.), balanced normal chow (for 8 w.); all urine samples were analyzed using a combination of liquid chromatography with tandem mass spectrometry and statistically tested non-parametrically using the Kruskal-Wallis test; data are presented as median ± interquartile range (IQR); extreme outliers beyond 3xIQR have been excluded.

24 h urine collections were sampled every week in order to assess the renal excretion of HCys and HCA. The amount of excreted HCys steadily increased in all animals during the 8-week-long period on B-vitamin deficient chow (Figure 3). At week 9, mean urinary HCys (male + female) of about 20.3 µg (≈0.15 µmoL) was excreted by WT mice and about 97.2 µg (≈0.72 µmoL) by the KI animals in 24 h. Similar to the findings in the serum, *App^NL-G-F^* KI mice displayed significantly higher urinary HCys amounts compared to WT (week 9 (male + female): *p* = 0.001). However, at baseline level and after normalization, HCys excretion did not differ between WT and KI animals (week 1: *p* = 0.228; week 10: *p* = 0.095). Furthermore, no sex-related effect was observed. Normalization of HCys excretion was achieved immediately after the return to control chow. Baseline levels were restored within one week.

**Figure 4:**
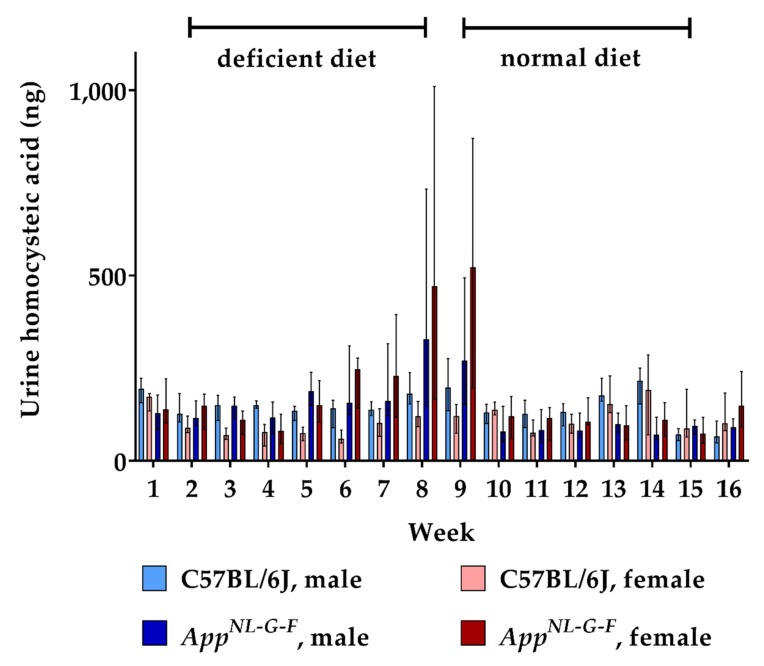
Urinary homocysteic acid levels in C57BL/6J wild type and *App^NL-G-F^* knock-in mice, data are depicted for males and females separately for experimental week 1–17: baseline measurement (w. 1), diet deficient in vitamin B6, B12 and folate (for 8 w.), balanced normal chow (for 8 w.); all urine samples were analyzed using a combination of liquid chromatography with tandem mass spectrometry and statistically tested non-parametrically using the Kruskal-Wallis test; data are presented as median ± interquartile range (IQR); extreme outliers beyond 3xIQR have been excluded.

Feeding the deficient diet also resulted in an increased urinary excretion of HCA in *App^NL-G-F^* KI mice, but not in WT control mice (Figure 4). Consequently, urinary HCA amounts were significantly higher in hyperhomocysteinemic KI mice (week 9: *p* = 0.002). As for HCys, we did not detect a consistent sex-dependent impact on the renal clearance of HCA. The conversion from B-vitamin deficient chow to normal chow decreased HCA excretion in the KI animals to baseline level within two weeks. Overall, dietary impact on urinary HCA was not as high as in the case of HCys.

### 2.3. Brain Tissue Analysis

**Figure 5:**
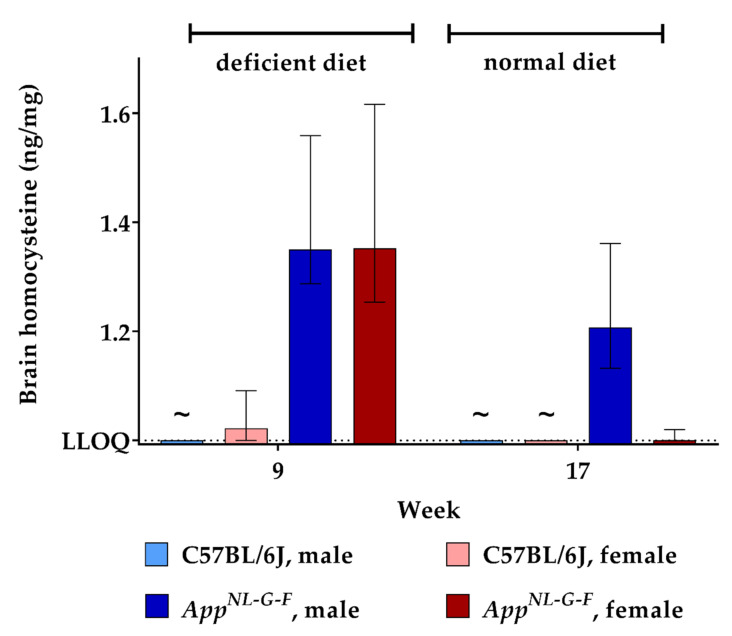
Homocysteine levels in brain tissue in C57BL/6J wild type and *App^NL-G-F^* knock-in mice, data are depicted for males and females separately for experimental week 1–17: baseline measurement (w. 1), diet deficient in vitamin B6, B12 and folate (for 8 w.), balanced normal chow (for 8 w.); all brain samples were analyzed using a combination of liquid chromatography with tandem mass spectrometry and statistically tested non-parametrically using the Kruskal-Wallis test; data are presented as median ± interquartile range (IQR); extreme outliers beyond 3xIQR have been excluded; ~indicates levels below the lower limit of quantification (LLOQ).

Murine brains were analyzed for HCys and HCA at week 9 and week 17 of the experiment. As no animals were sacrificed at the beginning of the study, there were no cerebral baseline measurements for HCys and HCA. Data obtained after feeding control chow for eight weeks (week 17) therefore served as a control in this case. Feeding with the B-vitamin deficient diet for eight weeks resulted in higher cerebral HCys in both WT and KI mice (week 9) compared to control levels (week 17). Hyperhomocysteinemic *App^NL-G-F^* mice displayed significantly higher levels than the C57BL/6J control (*week 9*; *p* = 0.001). There was no statistically significant sex-dependent impact on cerebral HCys at week 9. Although cerebral HCys was reduced in all groups after the control chow period (week 17), male KI mice still displayed relatively high levels compared to females and wild type mice. Results for HCys in brain tissues are presented, in part, in a descriptive manner, because all measured WT samples after eight weeks on control chow (week 17) had very low levels, below the lower limit of quantification (<LLOQ) of our LC-MS/MS method, which is 1.000 ng/mg tissue (Figure 5).

**Figure 6:**
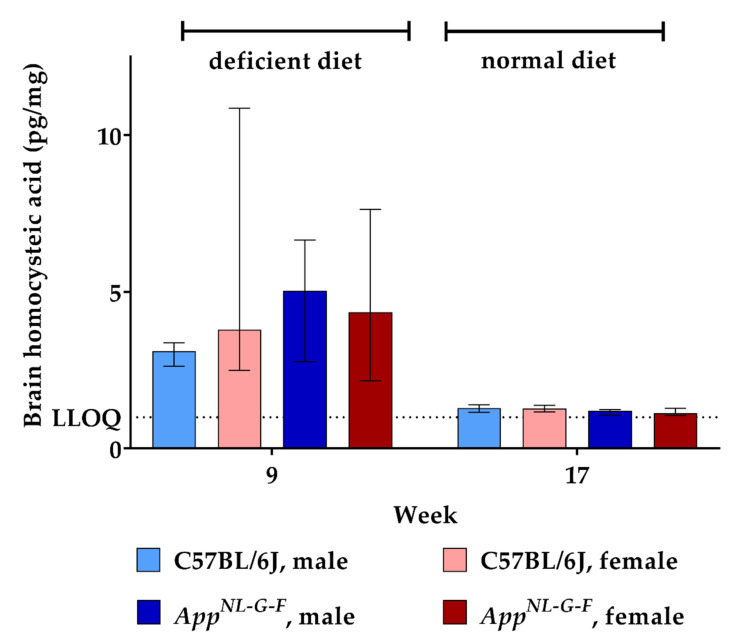
Homocysteic acid levels in brain tissue in C57BL/6J wild type and *App^NL-G-F^* knock-in mice, data are depicted for males and females separately for experimental week 1–17: baseline measurement (w. 1), diet deficient in vitamin B6, B12 and folate (for 8 w.), balanced normal chow (for 8 w.); all brain samples were analyzed using a combination of liquid chromatography with tandem mass spectrometry and statistically tested non-parametrically using the Kruskal-Wallis test; data are presented as median ± interquartile range (IQR); extreme outliers beyond 3xIQR have been excluded.

Similar to what we found for brain HCys levels, feeding the deficient diet increased brain HCA (week 9) compared to control levels (week 17), as illustrated in Figure 6. The difference between KI and WT mice did not reach statistical significance here (week 9). Also, no sex-related differences were observed between males and females. Another eight weeks on control chow decreased cerebral HCA concentrations to about 1.2 pg/mg tissue in the mice. In the case of HCA, we were able to assess cerebral levels at both week 9 and week 17 quantitatively, due to a more sensitive method reaching an LLOQ of 1.000 pg/mg tissue.

### 2.4. Correlation

Murine samples from different biological matrices were collected during the course of the current study in order to assess bioavailability and kinetics of HCys and HCA. After the induction of a hyperhomocysteinemic state through B-vitamin deficiency (week 9), we detected a significant positive correlation for HCys between its serum and urine levels (Table 1). Serum HCys levels were also positively correlated with HCys levels in cerebral tissues. For HCA, serum and urine correlation data indicated the same type of effects as for HCys (Table 1). Serum HCA also correlated positively with cerebral HCA concentrations. Furthermore, serum HCys correlated positively with serum HCA and urinary HCys correlated positively with urinary HCA. However, no significant correlation was found between cerebral HCys and cerebral HCA. In summary, we observed the overall relation that low HCys and HCA at baseline level (at the beginning or the end of the study) did not show any correlation, whereas a positive correlation was found after the onset of hyperhomocysteinema.

## 3. Discussion

In the current in vivo kinetic study, frequent sampling of murine matrices and subsequent LC-MS/MS analysis of HCys and HCA was carried out to clarify whether the AD-like genotype in the *App^NL-G-F^* KI mouse model had an impact on the hyperhomocysteinemic state. Furthermore, potential sex-specific differences, the bioavailability of HCys and HCA in the brain and correlations between serum, urine and cerebral tissues were investigated. In similar previous experiments, hyperhomocysteinemia was mostly induced chemically and kinetic parameters were investigated within a maximum of 24 h [30,39,40,41,42]. In contrast, we conducted a long(er)-term kinetic study by inducing chronically elevated levels of HCys and HCA using a dietary approach and investigating both increases and decreases in these endogenous metabolites over 17 weeks.

### 3.1. Genotype Effects

Amyloid pathology is a crucial and initial hallmark of AD, occurring many years before the onset of cognitive symptoms [43,44]. We used the novel *App^NL-G-F^* KI mouse model in order to simulate the AD-like amyloid pathology more realistically, compared to APP-based transgenic models [10]. Numerous studies in the field report an exacerbation of Aβ accumulation and deposition induced by elevated levels of HCys [45,46,47,48,49,50] or HCA [51,52]. As the majority of in vivo studies focused on HCys levels and its consequences for AD hallmarks, vice versa, the impact of amyloid pathology on HCys levels has been poorly investigated. In our study, we observed a B-vitamin deficiency-triggered elevation of HCys and HCA, which proved to be significantly higher in *App^NL-G-F^* KI mice in comparison to C57BL/6J WT mice. This is in accordance with previous findings by Bernardo and colleagues [53], who investigated HCys plasma levels in female, transgenic APP-overexpressing mice. An impact of the AD-like genotype on HCys metabolism was proposed, because it obviously induced higher HCys in both the control diet group and the methyl-donor deficient diet group [53]; hippocampal cell death was reported in transgenic but not in WT mice [53,54]. A similar genotype effect was observed in ArcAβ transgenic mice [55], where a diet-induced hyperhomocysteinemia occurred in the transgenic mice in contrast to WT control mice. This genotype effect might possibly be explained by reactive oxygen species that are part of the amyloid pathology in AD [56]. Oxidative stress is suggested to deplete folate [57], which plays an essential role in the context of the HCys remethylation cycle. Consequently, oxidative stress and the subsequent lack of folate could be the reason for the occurrence of the hyperhomocysteinemic state [58]. In order to elucidate mechanistical details, future experiments will be helpful. As summarized by Hasegawa and Ukai, the oxidative metabolite HCA is also suggested to be increased by oxidative stress via amyloid pathology [59]. However, our data do not confirm a significant elevation of serum HCA compared to the WT control. Also, the HCA/HCys ratio was not increased in the *App^NL-G-F^* KI model.

### 3.2. Sex-Specific Effects

WT males tended to display higher HCys and HCA in serum and urine than females after eight weeks on the deficient chow. This is translationally in accordance with the observation that men display higher HCys levels than women [60,61], a difference that is suggested to be the result of hormonal effects [62,63]. As summarized by Cohen and colleagues [4], a lower transsulfuration rate and higher creatine metabolism in men might also lead to the sex-related divergence in HCys levels. However, the aforementioned tendency fades out by also taking the findings in the KI animals into account. In total, no consistent sex-dependent effect was detected during the course of our study. According to this, other clinical [15] and preclinical [40] findings do not confirm significant sex-related effects. Others even detected higher HCys in female mice than in males [64], so all things considered, the evidence is equivocal in this topic. A limitation of our study is that no quantification of the amyloid pathology in the KI mice has been undertaken. Previous investigations in the *App^NL-G-F^* model did not indicate a sex difference in the Aβ plaque load [65].

### 3.3. Brain HCys and HCA

Levels of HCys and HCA, especially the bioavailability in the brain, which is essential information in the context of neurodegeneration, have not yet been characterized in the novel *App^NL-G-F^* KI mouse model for AD. Data for HCys and HCA were reported previously in human CSF [51,66,67,68]. However, we sampled cerebral tissues in the current study, because murine CSF would not have delivered enough volume to apply our LC-MS/MS methods. We detected HCys levels at about 1 ng/mg tissue in the *App^NL-G-F^* brains and thus, within the range of 0.135 ng/mg and 44 ng/mg that has been reported previously [30,69,70,71,72]. However, levels are hardly directly comparable between the in vivo studies, because different rodent models, as well as varying strategies to induce hyperhomocysteinemia, were used. Currently, we do not know the reason for the lower decrease in HCys in male KI mice on control chow (week 17). In combination with the poor correlation, we observed between HCys and HCA in cerebral tissues, this indicates that the underlying HCys metabolism in the brain, including the formation of HCA, might differ from the other matrices. HCA levels in our mice were detected at about 5 pg/mg tissue (week 9), which is not in accordance with one of the rare previous publications on HCA [52]. Hasegawa and colleagues reported 2000-fold higher HCA values for cerebral tissues and, in a subsequent publication, considered these relatively low [73]. Nevertheless, we agree with Hasegawa that AD pathology seems to significantly elevate HCys, but not HCA levels, in the CNS compared to non-AD controls [51].

### 3.4. HCys and HCA in Other Biological Matrices

HCys serum concentrations beyond 15 µmoL/L (≈2030 ng/mL) were considered high and referred to as hyperhomocysteinemia [74]. By feeding a diet deficient in vitamin B6, B12 and folate, we induced elevated levels of HCys beyond 15 µmoL/L and therefore, a hyperhomocysteinemic state in our mice. HCA concentrations were much lower than HCys, because oxidation of the latter is only possible in the free thiol form, which accounts for merely 1% [14].

In this study, we also focused on the correlation between HCys and HCA, as well as on the levels in different biological matrices. There was a significant correlation between elevated serum HCys and HCA in the mice after eight weeks on deficient chow, but not for baseline levels at the beginning or at the end of the study. This lack of correlation was also previously reported for plasma levels and might be explained by different levels of oxidative stress and its impact on the spontaneous oxidation of HCys to HCA [36,75,76]. Hasegawa reported an inverse correlation between blood HCA and urinary HCA and suggested that a reduced renal excretion results in elevated blood levels and subsequent cognitive impairment in patients [73]. Our murine HCA data, indicating increasing urinary excretion at increasing serum levels, did not confirm this relation and were detected at lower levels [52,73]. Recently, lower levels were also measured in human serum by conducting LC-MS/MS analytics [68,77]. In humans, urinary excretion of unchanged HCys is considered minimal due to a high extent of reabsorption in renal tubules [3]; about 6 µmoL (≈811 µg) HCys are excreted per day [78]. According to our results, lack of essential HCys-reducing B-vitamins led to a more than 10-fold elevated excretion of HCys in the kidney in both experimental mice and humans [3].

## 4. Materials and Methods

### 4.1. Animals, Diets and Study Design

All animal experiments were carried out according to the DIRECTIVE 2010/63/EU and the regulations of GV-SOLAS, approved by the local Ethics Committee for Animal Research in Darmstadt, Germany (approval number: F152/1011; approval date: 31.07.2017) and based on the ARRIVE-Guidelines.

A total of 80 mice were included in this study, equally consisting of C57BL/6J wild type (20 males, 20 females) and age-matched *App^NL-G-F^* knock-in mice (20 males, 20 females; C57BL/6J background). Wild type animals (WT) were obtained from Charles River Wiga GmbH (Sulzfeld, Germany) and knock-in (KI) animals were kindly provided by the RIKEN Center for Brain Science (Saitama, Japan) and further bred at mfd Diagnostics GmbH (Wendelsheim, Germany). All animals were allocated to their home cages (Green Line, Tecniplast, Hohenpeissenberg, Germany) according to a randomization list (https://www.random.org/) and were housed pairwise at constant temperature (mean: 22.7 °C) and humidity (mean: 55.4%) conditions under a 12/12 h dark/light cycle (lights on at 7:00 am).

**Figure 7:**
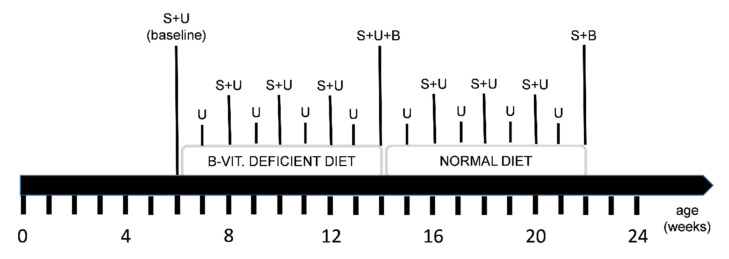
Time line of the study course, diet regimen and sampling points: S = serum sampling, U = 24-h urine sampling in metabolic cages, B = brain sampling: one half of the animals was euthanized in the middle (experimental week 9) and the other half at the end of the study (experimental week 17); feeding a diet deficient in vitamin B6, folate (B9) and B12 for 8 weeks was followed by a normal diet period for another 8 weeks.

After baseline serum and urine sampling, all mice received a special diet deficient in vitamin B6, B9 (folate) and B12 for eight weeks. Subsequently, half of the animals were euthanized in order to collect the brain tissue. The other half of the animals returned to a balanced control chow (normal diet) for another eight weeks in order to investigate a potential normalization of the levels of HCys and HCA. Potential side effects that might be induced by over-supplementation with B-vitamins are not further investigated or described here [79,80,81,82,83,84,85]. Sampling steps were carried out frequently in order to collect different biological matrices. The exact study course is illustrated in Figure 7.

Because mice are prone to coprophagia, the antibiotic sulfathiazole sodium (Sigma-Aldrich, Taufkirchen, Germany) had been added to the deficient diet in order to prevent folate synthesis by gut bacteria [45]. Both the B-vitamin deficient and the control diet were obtained from (Ssniff-Spezialdiäten GmbH, Soest, Germany). Table 2 indicates the exact composition of these diets, based on the AINM93M chow. Each animal received four gram of diet per day and water ad libitum. All mice were weighed every week and scored for body condition twice a week in order to monitor the nutritional status.

### 4.2. Sample Collection

Before the start of the experimental diet, serum and urine were collected from the mice at the age of 6 weeks in order to assess baseline levels of HCys and HCA. In accordance with the national animal welfare guidelines of the GV-SOLAS, we collected blood (170 µL per 25 g mouse) every two weeks during the study course. For the purpose of blood collection, we anaesthetized the mice using isoflurane (Piramal Critical Care, Hallbergmoos, Germany) and subsequently punctured the retrobulbar vein with a glass capillary (Brand GmbH + Co KG, Wertheim, Germany). Blood was collected in serum tubes containing a clotting factor (Sarstedt Microvette 200 Z, Nümbrecht, Germany) and coagulated for 15–30 min. Subsequent centrifugation (3200 g; 4 °C; 15 min) provided serum. Additionally, urine from each mouse was sampled every week using metabolic cages (Tecniplast, Hohenspeissenberg, Germany). Serum and urine were immediately frozen on dry ice and stored at −80 °C for further biochemical analysis. Volumes of the urine samples were documented in order to determine the absolute excretion of HCys and HCA in 24 h. At the age of 14 weeks, resp. after 8 weeks on B-vitamin deficient diet, half of the animals was euthanized by cervical dislocation and brains were harvested. After the removal of cerebellum and olfactory bulbs, the hemispheres were divided, weighed and frozen in liquid nitrogen for subsequent analysis of HCys and HCA. Afterwards, the remaining animals returned to control chow for 8 weeks. At the end of the study, the remaining animals were euthanized, and brains were sampled and processed as described before.

### 4.3. Biochemical Analysis

The determination of HCA in serum and urine samples was performed as described in detail [38,77], using a combination of protein precipitation (ice-cold acetonitrile) and solid phase extraction (tabless Strata X AW SPE columns (33 µm, 30 mg/1 mL, Phenomenex, Aschaffenburg, Germany) and the automated sample preparation system Extrahera (Biotage, Uppsala, Sweden)) for sample preparation followed by an LC–MS/MS analysis applying a combination of a HILIC separation (Luna 3 µm HILIC 200 Å 100 × 2 mm column in combination with a KrudKatcher in-line filter (both Phenomenex, Aschaffenburg, Germany)) and tandem mass spectrometry. As the concentration of HCA in murine samples is higher than in human samples, lower sample volumes were used for murine samples and PBS was added to achieve a total sample volume of 200 µL. Thereafter, the samples were processed as described [77]. HCys in serum and urine samples was analyzed using protein precipitation (methanolic TCEP solution) in combination with reversed phase chromatography (Luna Omega 1.6 μm Polar C18 100 × 2.1 mm column in combination with a respective pre column (both Phenomenex, Aschaffenburg, Germany)) and tandem mass spectrometry as described in detail [38]. As we had not analyzed brain samples for HCA or HCys in our lab before, the established methods were adapted to the new matrix. The first step was the homogenization of the brain samples using a weight-dependent volume of a mixture of water and ethanol (*v*:*v*–75:25) and a swing mill (Mixer Mill MM 400, Retsch, Haan, Germany) generating a homogenate with a tissue concentration of 0.2 mg/mL. A sample volume of 200 µL homogenate was used for the determination of HCA using the same sample preparation protocol and LC-MS/MS method as for serum and urine. The determination of HCys in brain tissue homogenate using reversed phase chromatography was not possible as interferences in the chromatogram occurred making the quantification of HCys in these samples difficult. Therefore, a hydrophilic interaction liquid chromatography (HILIC) method was applied using the same column as for HCA and a mixture of water, acetonitrile, and 0.1 M ammonium acetate solution (88:10:2, *v*/*v*/*v*) as solvent A, and acetonitrile containing 0.1% formic acid as solvent B. For separation, a gradient program was used at a flow rate of 0.75 mL/min. The initial buffer composition 3% (A)/97% (B) was held for 0.25 min and then within 2.25 min, linearly changed to 50% (A)/50% (B) and held for 1.00 min. Subsequently, the composition was linearly changed within 0.1 min to 3% (A)/97% (B) and then held for another 3.4 min. The total running time was 7 min, and the injection volume was 2.5 µL. The sample preparation protocol was identical to the procedure for the determination of HCys in serum and urine.

The LC-MS/MS system for the determination of HCA in serum, urine and brain tissue as well as for the determination of HCys in serum and urine consisted of a triple quadrupole mass spectrometer QTRAP 6500+ (Sciex, Darmstadt, Germany) equipped with a Turbo Ion Spray source and an Agilent 1290 Infinity LC-system with binary HPLC pump, column oven and autosampler (Agilent, Waldbronn, Germany). The LC-MS/MS system for the determination HCys in brain tissue consisted of a triple quadrupole mass spectrometer QTRAP 5500+ (Sciex, Darmstadt, Germany) equipped with a Turbo Ion Spray source and an Agilent 1200 LC-system with binary HPLC pump, column oven (Agilent, Waldbronn, Germany) and an HTC PAL autosampler (CTC Analytics, Zwingen, Switzerland). Data acquisition was done using Analyst Software 1.7.1 and quantification was performed with MultiQuant Software 3.0.3 (both Sciex, Darmstadt, Germany), employing the internal standard method. Calibration curves were calculated by linear regression with 1/x weighting. Acceptance criteria and quality assurance measures have been applied as previously described [77].

### 4.4. Statistical Analysis

Prior to the study, a statistical power calculation was conducted in order to estimate the adequate number of mice for the experiments (http://www.biomath.info/power/). For the statistical analysis of the data, we used IBM SPSS Statistics 26 (Ehningen, Germany). We conducted an outlier analysis to detect extreme outliers (more than threefold the interquartile range). Shapiro-Wilk test indicated that a Gaussian distribution could not be assumed for various data sets. Consequently, the non-parametric Kruskal–Wallis test (+ Bonferroni adjustment for *p*-values) and Spearman rank correlation test were applied. A *p* value lower than 0.05 was considered statistically significant. Data were depicted as median ± interquartile range (IQR), separately for males and females, using GraphPad Prism 7 software (San Diego, CA, USA).

## 5. Conclusions

1. In this exploratory in vivo kinetic study, we observed a significant genotype effect on HCys, but not HCA, in hyperhomocysteinemic *App^NL-G-F^* KI mice compared to the age-matched C57BL/6J WT control and therefore, confirmed a potential causal contribution of the AD-like pathology to hyperhomocysteinemia.
2. Our data did not indicate a consistent sex-related effect on the hyperhomocysteinemic state and therefore did not meet the expectation that levels are generally higher in males.
3. HCys and HCA were detected in cerebral tissues of WT and KI mice, which is a crucial condition for the potential contribution to pathologies in the context of neurodegenerative disorders. The consumption of a B-vitamin deficient diet resulted in increased cerebral levels of HCys and HCA.
4. In hyperhomocysteinemic mice, serum HCys and HCA levels correlated positively with both cerebral concentrations and urinary amounts.

To our knowledge, this is the first in vivo kinetic study that considers HCA, which might be the actual culprit in terms of neuronal damage, additionally to HCys. The observed genotype impact in the *App^NL-G-F^* KI mouse model for AD suggests that AD pathology affects HCys levels and indicates a synergistically exacerbating effect of B-vitamin deficiency and AD pathology on hyperhomocysteinemia. The immediate normalization of HCys and HCA after the supply of relevant B-vitamins in a balanced diet, strengthens the idea of a B-vitamin intervention in order to interrupt HCys-triggered pathologic processes. Additional studies will have to be performed to corroborate the previously suggested potential as a preventative treatment option for AD, which might be effective and easily feasible.

## Figures and Tables

**Table 1:** Spearman’s rank correlation analysis of hyperhomocysteinemic mice at week 9 (after 8 weeks on deficient diet).

Correlation	Correlation Coefficient	Sig. (2-Tailed)
Serum-urine (HCys)	0.771	<0.001
Serum-brain (HCys)	0.735	<0.001
Serum-urine (HCA)	0.675	<0.001
Serum-brain (HCA)	0.521	0.001
HCys-HCA (serum)	0.660	<0.001
HCys-HCA (urine)	0.860	<0.001
HCys-HCA (brain)	0.142	0.509

**Table 2:** Exact composition of the control (normal) diet and the B-vitamin deficient diet.

	Control	Deficient
Casein	140.0	140.0
Corn starch	355.6575	345.6920
Maltodextrin	155.0	155.0
Sucrose	100.0	100.0
Dextrose	100.0	100.0
Cellulose	50.0	50.0
Mineral premix	35.0	35.0
Vitamin premix (w/o B-vitamins)	10.0	10.0
Soybean oil	19.0	19.0
Coconut oil	9.0	9.0
Corn oil	22.0	22.0
L-Cystine	1.8000	1.8000
Tert-butylhydroquinone	0.0080	0.0080
Choline bitartrate, 41%	2.5000	2.5000
Pyridoxine-HCl (Vit. B6)	0.0070	——
Cyanocobalamin, 0.1% (Vit. B12)	0.0250	——
Folic acid, 80%	0.0025	——
Sulfathiazole sodium	——	10.0000
Sum	1000	1000

## Data Availability

The data presented in this study are available on request from the corresponding author.

## Abbreviations

AD	Alzheimer’s disease
HCys	Homocysteine
HCA	Homocysteic acid
WT	Wild type
KI	Knock-in
LC-MS/MS	Liquid chromatography with tandem mass spectrometry
HPLC	High-performance liquid chromatography
HILIC	Hydrophilic interaction liquid chromatography
LLOQ	Hydrophilic interaction liquid chromatography
GV-SOLAS	Gesellschaft für Versuchstierkunde / Society of Laboratory Animal Science
